# Feasibility, effectiveness, and acceptability of an afternoon-evening sleep schedule in older nightshift workers

**DOI:** 10.1093/sleepadvances/zpae010

**Published:** 2024-02-07

**Authors:** Laura K Barger, Yuan Zhang, Heidi M Lammers-van der Holst, Davina Snoep, Audra S Murphy, Brian Desnoyers, Jeanne F Duffy

**Affiliations:** Division of Sleep and Circadian Disorders, Department of Medicine, Brigham Women’s Hospital, Boston, MA, USA; Division of Sleep Medicine, Harvard Medical School, Boston, MA, USA; Solomont School of Nursing, Zuckerberg College of Health Sciences, University of Massachusetts Lowell, Lowell, MA, USA; Division of Sleep and Circadian Disorders, Department of Medicine, Brigham Women’s Hospital, Boston, MA, USA; Division of Sleep Medicine, Harvard Medical School, Boston, MA, USA; Division of Sleep and Circadian Disorders, Department of Medicine, Brigham Women’s Hospital, Boston, MA, USA; Division of Sleep and Circadian Disorders, Department of Medicine, Brigham Women’s Hospital, Boston, MA, USA; Division of Sleep and Circadian Disorders, Department of Medicine, Brigham Women’s Hospital, Boston, MA, USA; Division of Sleep Medicine, Harvard Medical School, Boston, MA, USA

**Keywords:** shiftwork, nightshift, sleep, circadian misalignment, healthcare workers, older adults

## Abstract

**Study Objectives:**

To explore the feasibility, effectiveness, and acceptability of an afternoon-evening sleep schedule in older (age 50–65 years) nightshift workers.

**Methods:**

We used a three-part strategy: a screening survey to identify individuals who said they could adopt an 8-hour afternoon-evening sleep schedule; a field study where daily diary and actigraphy data were collected during a baseline week and intervention week, with randomization to self-selected sleep, 8-hour afternoon-evening time in bed (TIB), or 8-hour self-selected TIB; and follow-up focus groups to understand the acceptability of the intervention.

**Results:**

Gender (*p* < 0.001), Hispanic ethnicity (*p* = 0.023), the care of children (*p* = 0.014), and chronotype (*p* = 0.012), predicted the reported ability to spend 8 hours in bed in the afternoon-evening. Participants assigned to the 8-hour self-selected and 8-hour afternoon-evening groups significantly increased their TIB and sleep duration compared to baseline (*p* < 0.05), while the control group did not. Although spending 8 hours in bed was feasible for the participants during the study, focus group discussions indicated participants would not continue an 8-hour TIB schedule after the study due to family responsibilities and other activities of daily living.

**Conclusions:**

Spending 8 hours in bed between successive night shifts, initiated at both a self-selected time and in the afternoon-evening, increased the sleep duration of older shiftworkers, but most would not continue such a schedule on their own. Additional research is needed to find countermeasures for the reduced sleep duration experienced by most shiftworkers that are not only effective, but also compatible with shiftworkers’ lifestyles.

**Statement of Significance**

This research describes the feasibility, effectiveness, and acceptability of an afternoon-evening sleep strategy for lengthening sleep duration between successive night shifts in older workers. We found that despite the effectiveness of the afternoon-evening sleep strategy to increase sleep duration, participants deemed it unsustainable due to prioritizing childcare, family responsibilities, and other daily activities. Future research should develop sleep strategies that are compatible with the lifestyle of older shift workers and investigate the impact of the strategies on worker performance and satisfaction.

## Introduction

Night shift work is challenging. Nightshift workers must remain alert and perform optimally when their circadian clock is programmed for sleep, and then sleep when their circadian clock is programmed for wake. Not only is the ability to stay alert and perform well dependent on the time of day, but the quality and quantity of sleep exhibit circadian variation such that sleep during the day is shorter and of poorer quality than sleep during the night [1–3]. Alertness and performance also heavily depend on sleep quality and duration. Therefore, the circadian misalignment associated with nightshift work causes decrements directly and indirectly via its effects on sleep [4–6]. These adverse effects of night work are exacerbated in older workers compared to young workers, mainly due to their decreased ability to sleep during the day [7–10].

Most interventions to improve night shift performance focus on strategies to shift the timing of the circadian clock, and/or acutely increase alertness during work [11–15]. The timing of nightshift workers’ sleep is often overlooked with these strategies. Unlike day workers who typically arise only an hour or two before starting work, nightshift workers usually sleep in the morning shortly after finishing their shift, thus awakening eight or more hours prior to reporting to their next night shift. Consequently, nightshift workers are beginning work with a greater amount of homeostatic sleep pressure due to prolonged wakefulness, which itself may also degrade alertness and performance during night shifts [16].

In a previous study of healthy older adults working simulated night shifts in the laboratory, we randomized individuals to spend 8 hours in bed in the afternoon-evening or to sleep ad lib at home between their simulated shifts in the laboratory. Afternoon-evening sleepers had significantly longer and less disrupted sleep as assessed via actigraphy than participants who slept ad lib, and they also showed better on-shift alertness and performance [11]. However, the participants were not actual shift workers, and it was unknown whether this sleep timing intervention would be feasible, effective, and acceptable in actual older night shift workers in the “real world,” where workers have family and caregiver responsibilities, commutes, and other actives of daily living. Therefore, we conducted a three-pronged approach to explore this, centered on a randomized clinical trial of an 8-hour afternoon-evening sleep timing countermeasure.

## Materials and Methods

We recruited shift workers to complete an online survey that served as a screening questionnaire for the field trial. Each potential participant completed the screening survey to provide demographic data and determine their eligibility. We restricted field trial participants to healthcare workers aged 50–65 years. Other inclusion criteria were regularly working 8-hour night shifts (with shifts starting from 10 pm to midnight), at least four times per month, and the ability to arrange their off-duty schedule to spend eight consecutive hours between 12:00 and 22:00 in bed trying to sleep between successive night shifts. Participants were excluded if they regularly took prescription or over-the-counter medications known to affect sleep or alertness.

**Figure 1.**
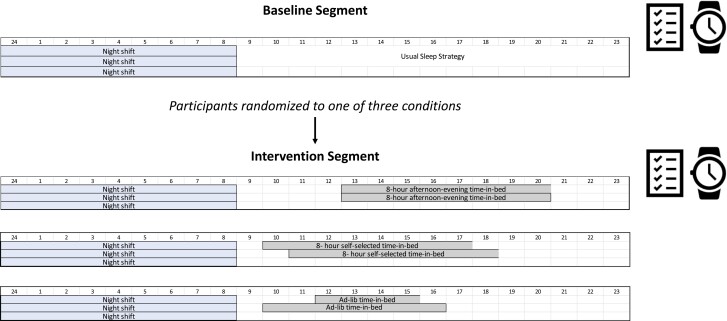
Schematic of study protocol. Participants began with the baseline segment of at least three consecutive night shifts during which they followed their usual sleep strategies. This was followed by an intervention segment during which participants were randomized to one of the three time-in-bed strategies between at least three consecutive night shifts: 8-hour afternoon-evening time-in-bed, 8-hour self-selected time-in-bed, or ad-lib time-in-bed. Time-in-bed was collected via sleep diary and sleep was estimated with actigraphy.

Each participant was monitored over two separate work weeks (segments), with at least two consecutive night shifts planned in each week. (Figure 1) The first segment served as the baseline for all participants, during which they were instructed to continue their usual sleep strategies. After the first night shift of the second (intervention) segment, participants were randomized into one of three groups: ad-lib time in bed (TIB) (control); 8-hour afternoon-evening TIB; or 8-hour self-selected TIB. The control group was not given any instruction about the timing or duration of their sleep. Participants in the 8-hour afternoon-evening TIB group were instructed to go to bed between 13:00 and 14:00 and to remain in bed for 8 continuous hours; those in the 8-hour self-selected TIB group were told to remain in bed for 8 continuous hours but were not given any instruction regarding the timing of the sleep episode. Participants were asked to keep the assigned schedule between all consecutive night shifts during the intervention segment.

TIB was assessed between each night shift with an electronic diary. Participants recorded the time they tried to fall asleep (“What time did you try to fall asleep?”) and the time they got out of bed (“At what time did you finally get out of bed?”), and the difference was defined as TIB.

Chronotype was assessed on the screening questionnaire using question 19 on the Morningness–Eveningness Questionnaire [17] with possible responses including: definitely a morning type, rather more a morning than evening type, rather more an evening than a morning type, definitely an evening type, and neither. The one-item assessment was used to reduce participant questionnaire burden.

Throughout both the baseline and intervention segment, each participant wore a MotionWatch8 (CamNtech Inc., Boerne, TX) to record activity. Sleep duration was estimated with MotionWare software (CamNtech Inc., Boerne, TX). Each day of actigraphy data was visually inspected and the diary times were used to guide the placement of sleep analysis windows [18].

To gain further understanding of the acceptability of the afternoon-evening sleep countermeasure, we conducted virtual focus groups with field trial participants. After completion, each participant was invited to take part in a 90–120-minute virtual focus group within 1–2 months following their study. Structured focus group discussions were guided with a pre-developed focus group guide. The primary topics included discussions about participants’ current night shift schedules, their sleep patterns and experiences between two consecutive shifts, before the first night shift, and after the last night shift, factors influencing their current sleep patterns, non-sleep activities between two consecutive night shifts, and their opinions on the acceptability of the sleep timing intervention. To enable scheduling and stimulate dynamic discussion in the virtual environment, each focus group was limited to 2–4 participants [19, 20]. One experienced investigator (YZ) and one research assistant (ASM) facilitated all focus groups by secure Zoom videoconferencing, with participants using a self-selected pseudonym on screen and in the conversation.

All study procedures were approved by the Partners Healthcare Human Research Committee. Each survey participant was shown an information sheet prior to opting in to complete the screening survey. Each participant in the field trial provided written informed consent for the field study and focus group before beginning the field trial. Study data (surveys and daily diaries) were entered by participants through an in-house software application on an iPad mini and remotely collected using REDCap electronic data capture tools hosted at Brigham and Women’s Hospital [21, 22].

### Statistical analysis

We used the screening survey data and performed logistic regression to describe demographic factors associated with the feasibility of the afternoon-evening sleep countermeasure for survey participants. Mixed models were used to analyze differences in participants’ TIB and sleep duration between baseline and intervention segments and among control and intervention groups in the field trial, and to discern interaction effects. Segment and group were fixed effects in the model and random effects were used for participants. Statistical analysis was conducted with SAS version 9.4 (SAS, Cary, NC) and alpha was set at 0.05.

Focus group discussions were automatically transcribed by Zoom and verified by a research assistant before data analysis. The two facilitators reviewed the focus group transcripts several times to sort the discussion topics into categories. A start list of codes and participants’ quotes was created by the experienced investigator (YZ), confirmed by the research assistant (ASM), and discussed by the research team. Using the start list, the transcripts were then reviewed and coded in Microsoft Word by two research assistants who had received a 2-hour intensive qualitative content analysis training. The content analysis was based on a hybrid approach that included a balance of deductive coding based on question categories in the focus group guide and inductive coding from themes that emerged from focus group discussions [23]. Discrepancies were resolved by the two facilitators and two research assistants through interpretive discussions, consensus building, and refinement of code definitions as needed. Focus group participants were emailed a list of final themes to review and approve.

## Results

**Figure 2.**
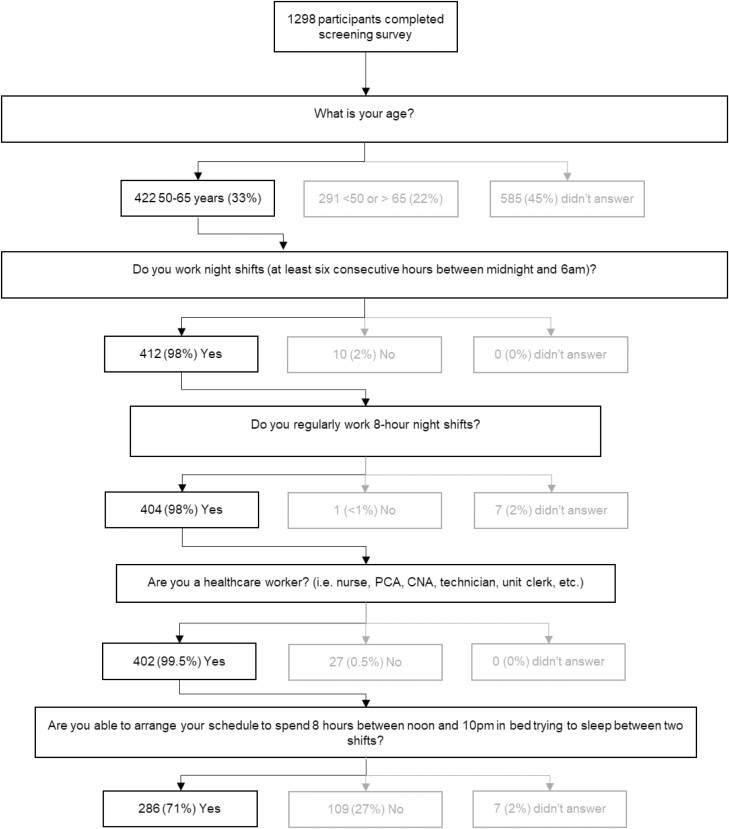
Self-reported feasibility of adhering to 8 hours of afternoon-evening sleep among survey respondents.

A total of 1289 shift workers completed the screening survey. Of those, 402 (33%) were in our targeted group of older (50–65 years) healthcare workers who worked 8-hour night shifts. The majority (286/402, 71%) indicated that an afternoon-evening sleep countermeasure would be feasible (Figure 2).

Gender (*p* < 0.001), Hispanic ethnicity (*p* = 0.023), the care of children (*p* = 0.014), and chronotype (*p* = 0.012), each significantly predicted the reported ability of the participants to arrange their schedule to spend 8 hours in bed in the afternoon-evening. Ninety percent of male respondents reported the ability for afternoon-evening sleep compared to only 63% of female respondents. Compared to non-Hispanics (67%), 91% of Hispanic respondents reported being able to arrange their schedule for afternoon-evening sleep (OR 3.54, 95% CI: 1.44 to 8.56). Having primary care of children reduced the likelihood of being able to schedule afternoon-evening sleep as compared to respondents who had no children under age 18 living at home (OR 0.34 [95% CI: 0.18 to 0.66]). Compared to neither type, morning chronotypes were more likely to report the ability to accommodate 8 hours in bed in the afternoon-evening (OR = 2.780, 95% CI 1.22 to 6.40).

### Field trial

Twenty-nine workers completed the field study, with 10 randomized to the control group, 10 to the 8-hour afternoon-evening TIB group, and 9 to the 8-hour self-selected TIB group. The majority were female (23/29, 79%) and reported their primary occupation as nursing (25/29, 86%). Mean age (± SD) was 56.5 ± 4.3 years. All were non-Hispanic and 86% were white. Demographic characteristics of the three groups were comparable (Table 1).

**Table 1.** Demographic Information for Field Trial Participants

		Control	8-hour self-selected sleep	8-hour afternoon-evening sleep
*n*		10	9	10
Age (mean ± SD)		54.6 ± 4.2	58.6 ± 2.9	58.3 ± 5.0
Gender	Female	90%	78%	70%
	Male	10%	22%	30%
Ethnicity	Hispanic or Latino	0%	0%	0%
	Not Hispanic or Latino	100%	100%	100%
Race	American Indian or Alaskan Native	10%	0%	0%
	Asian	0%	0%	0%
	Black or African American	0%	11%	20%
	Native American or Pacific Islander	0%	0%	0%
	White	90%	89%	80%
	More than one	10%		
	Prefer not to answer	0%	0%	0%
Education	12 years (high school graduate)	10%	11%	0%
	13–15 years (some college)	40%	33%	30%
	16 years (college graduate)	50%	33%	60%
	17 or more years (post-graduate degree)	0%	22%	10%
Marital status	Divorced or separated	10%	0%	0%
	Married or live with partner	80%	100%	80%
	Single, never been married	10%	0%	10%
	Widowed	0%	0%	10%
Childcare responsibility	I have primary responsibility	0%	0%	10%
	I share responsibility with another adult(s)	40%	22%	20%
	There are no children under 18 at home	60%	78%	70%
Household Income				
	$25 000–$34 999	0%	0%	10%
	$35 000–$49 999	10%	0%	0%
	$50 000–$74 999	30%	11%	10%
	$75 000–$99 999	10%	33%	0%
	$100 000–$149 999	30%	11%	50%
	Greater than $150 000	20%	44%	30%
Caregiver	Yes	20%	11%	10%
	No	80%	89%	90%
Second Job	Yes	10%	22%	10%
	No	90%	78%	90%
Years of night shift	Less than 1 year	10%	11%	0%
	1–5 years	10%	11%	10%
	6–10 years	10%	11%	10%
	11–24 years	30%	22%	50%
	25 + years	40%	44%	30%
Chronotype	Definitely a “morning” type	33%	0%	40%
	Rather more a “morning” than “evening”	0%	10%	10%
	Neither type	0%	20%	10%
	Rather more an “evening” than “morning”	22%	0%	30%
	Definitely an “evening” type	22%	60%	0%
	I don’t know how to answer	22%	10%	10%

^*^May not add to 100% due to rounding.

Not all participants worked the same number of consecutive night shifts in the baseline and intervention segments. To address this, we analyzed up to the first three nightshifts from each participant in each segment. Two participants had unusable actigraphy (one from the 8-hour afternoon-evening TIB group and one from the 8-hour self-selected TIB group) and three participants did not provide usable diary data (two from the 8-hour afternoon-evening TIB group and one from the 8-hour self-selected TIB group). The remaining participants contributed 129 days of actigraphy (*n* = 27) and 124 diary days (*n* = 26).

### Feasibility (Timing and duration of TIB)

**Figure 3.**
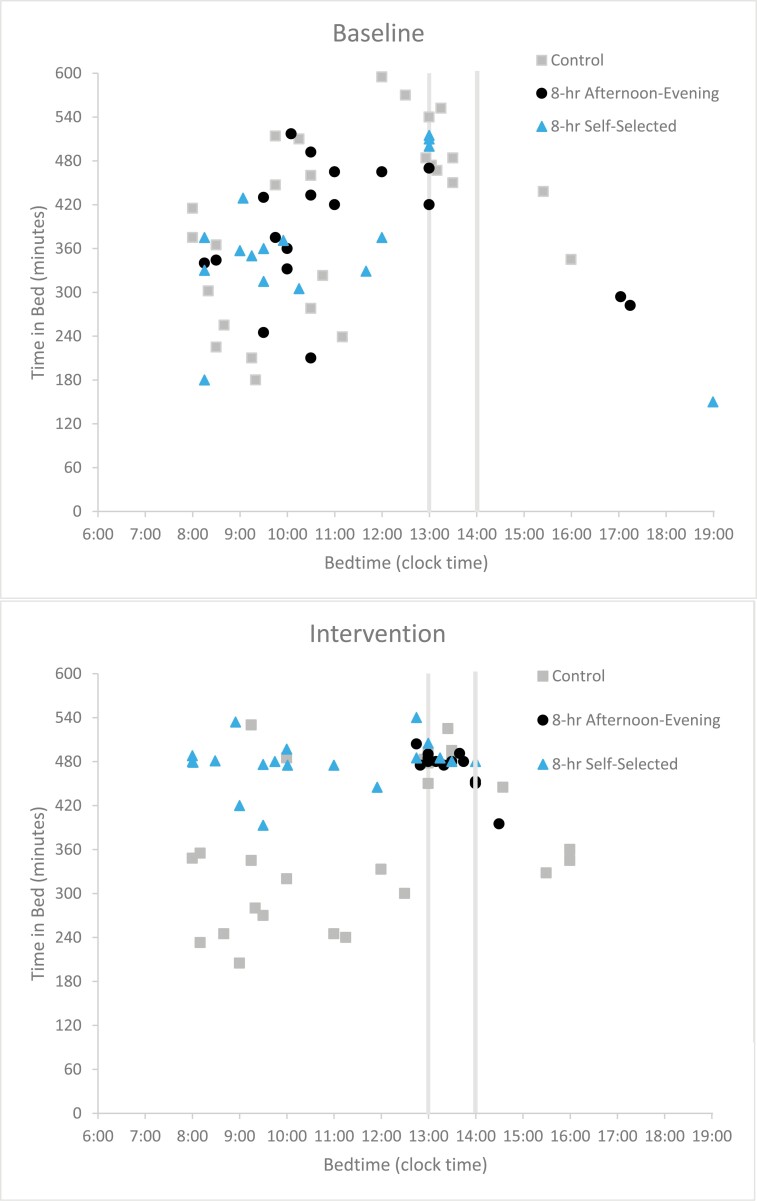
Timing and duration of all TIB episodes reported in participants’ sleep diaries by segment. In the intervention segment, those in the 8-hour afternoon-evening sleep group were instructed to go to bed between 13:00 and 14:00 (vertical lines), those in the self-selected group were instructed to spend 8 hours in bed at a time of their choosing, and those in the control group were given no instructions.

The timing and duration of time spent in bed between night shifts, as reported in the diaries, is illustrated in Figure 3. In the baseline segment, only 19% of all participants averaged at least 8 hours of TIB. Similarly, only 20% of the control group participants averaged 8 or more hours of TIB during the intervention segment. In the 8-hour afternoon-evening TIB and the 8-hour self-selected TIB groups, half (50%) of the participants averaged at least 8 hours of TIB during the intervention segment (68% of reported TIB episodes in the 8-hour afternoon-evening sleep condition and 63% TIB episodes in the 8-hour self-selected sleep condition). The mean start time of TIB episodes in the afternoon-evening sleep condition was (13:20 ± 0:30). The majority (17/19, 89%) of reported TIB episodes in the afternoon-evening sleep condition were at or after 13:00, as directed, with the two non-compliant TIB episodes starting at 12:45 and 12:50. The control group participants’ mean TIB start was 11:33 ± 2:31 and the participants in the self-selected 8-hour TIB group has a mean start time of 10:35 ± 2:04.

**Figure 4.**
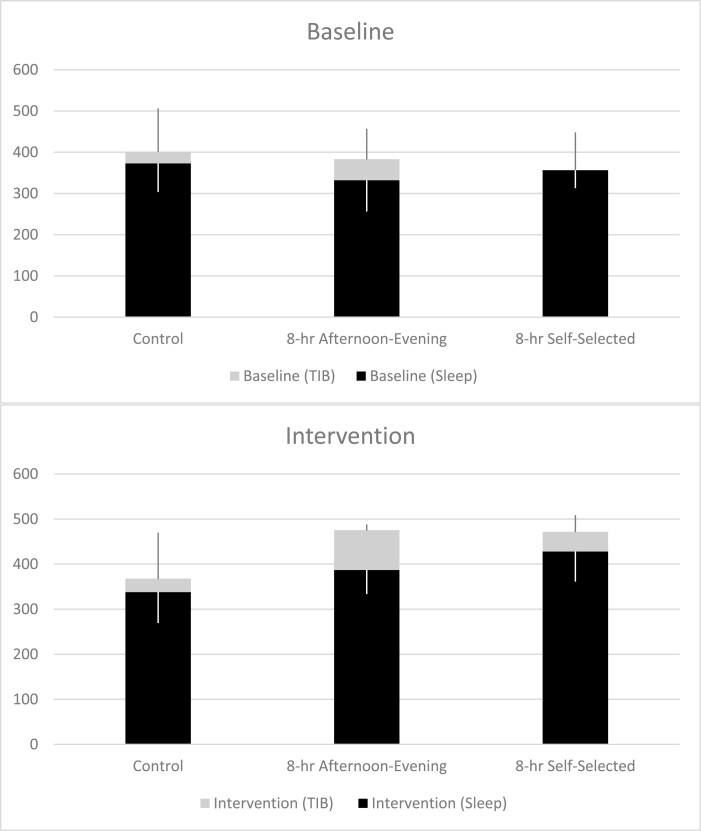
Mean (+SD) time in bed (gray) and sleep (black) for each group during the control and intervention segments. Time in bed was taken from the daily sleep diary and sleep duration was assessed from actigraphy. Note: We decided to use all available data in our analyses and representation of the data. Due to a mismatch in the number of participants that contributed diary and actigraphy data in the 8-hour Self-Selected Baseline group, the average TIB estimated from diary is similar to the actigraphy-estimated sleep.

Overall, TIB significantly increased in the intervention segment compared to the baseline segment (*p* = 0.0006) and there was a significant interaction between segment (baseline vs. intervention) and group (control, 8-hour afternoon-evening TIB, 8-hour self-selected TIB) (*p* = 0.0006). Compared to their baseline segment, both the 8-hour afternoon-evening TIB (*p* = 0.0004) and 8-hour self-selected TIB (*p* = 0.0003) groups increased their TIB by more than 90 minutes. TIB in the control group did not differ between the baseline and intervention segments. During the intervention segment, both the 8-hour afternoon-evening group (475.4 ± 12.8 minutes, *p* = 0.008) and the 8-hour self-selected group (471.9 ± 36.8 minutes, *p* = 0.010) had longer TIB than the control group (367.8 ± 102.1 minutes, Figure 4, Supplementary Table S1).

### Effectiveness (sleep duration)

Compared to the baseline segment, there was a trend for an increase in actigraphy-estimated sleep duration in the intervention segment (*p* = 0.052). There was a significant interaction between segment (baseline vs. intervention) and group (control, 8-hour afternoon-evening TIB, 8-hour self-selected TIB) in sleep duration (*p* = 0.014).

Compared to their baseline segment, both the 8-hour delayed afternoon-evening TIB (*p* = 0.043) and 8-hour self-selected TIB (*p* = 0.015) groups significantly increased their sleep duration. The average increase in sleep duration was 55.0 minutes in the afternoon-evening group and 71.5 in the 8-hour self-selected group. There was no difference in sleep duration in the control group between the baseline and intervention segments. In the intervention segment, sleep duration in the 8-hour self-selected TIB group was significantly increased (428.0 ± 66.7 minutes, *p* = 0.007) compared with the control group (338.0 ± 68.7 minutes), but sleep duration was not significantly different between the 8-hour delayed afternoon-evening TIB group (387.0 ± 53.2 minutes) and the control group (*p* = 0.11, Figure 4)

### Acceptablity

Nineteen field study participants (57.5 ± 3.8 years; 74% female, and 86% white) took part in one of the seven virtual focus groups (7/10 from the control group, 7/9 from the 8-hour self-selected TIB group, and 5/10 from the 8-hour afternoon-evening TIB group). Several themes emerged from the discussions.

Although most participants in the 8-hour afternoon-evening group and the 8-hour self-selected group complied with the study protocol of spending 8 hours in bed, focus group participants emphasized the challenges these schedules would present outside of the study. Participants explained that in general, they selected their sleep schedules based on family, social, and childcare responsibilities. One participant explained, “My husband always worked 3 to 11 so I had to pick them (children) up at 2:30 so I had to get to sleep right away to pick them up and to be up with them after school.” Another reported trying to coordinate sleep between night shifts to maintain “some family life . . . eating dinner, and you know watching a movie, playing games with them (family).”

Most participants preferred more than one sleep episode between nightshifts. They described the importance of using a split sleep schedule (morning sleep plus a nap before the night shift) to secure some time during the day for activities. One participant explained, “By breaking my sleep I have all my quality time during the day available, like you can go to my grandkids ball games . . . go visiting people . . . .”

Participants also reported that they were not used to staying in bed for 8 hours and it was difficult for them to do so. Many reported not sleeping the entire 8 hours and being frustrated at having to stay in bed, especially when other family members were home. One participant said, “My daughter came home from school, saying what’s wrong with mom why she’s still in bed.” Another reported that although she stayed in bed, she wasn’t sleepy and took on other tasks (e.g. paying bills in bed). Participants also expressed that the assigned 8 hours in bed affected their free time. They had to plan ahead because they knew they would not have the time they normally would for daily living tasks. One reported missing dinner with their family because of the required 8 hours in bed.

In addition, participants reported that staying awake between the time they got home after the night shift and the beginning of the 8-hour afternoon-evening TIB at 13:00 was challenging. One reported “being too tired in the morning to really do anything” in the interval between getting home in the morning and the 13:00 bedtime. Others reported a preference to sleep when their children were not home and said that delaying their sleep into the afternoon interfered with the need to pick up their children from school/bus stop in the early afternoon, cooking dinner, and/or spending evening time with their family. Others reported increased stress in worrying about getting enough sleep with the afternoon-evening sleep, “I’m having a hard time in bed, I’m tossing and turning . . . it’s five o’clock I’m looking at the clock and it’s 5:30 and I’m still trying to set my mind, because I’m like, crap, . . . I gotta get up at quarter 10, I gotta get ready for work and it’s already 5:30.…”

Many participants expressed skepticism at being able to maintain an 8-hour afternoon-evening TIB schedule beyond their participation in the study. They speculated that living alone, not having to work around anyone else’s schedule, or having additional support from a spouse/partner in taking care of the family in the afternoon might facilitate the delayed sleep schedule.

However, most focus group participants reported that although they had difficulty staying in bed for 8 hours, they actually obtained more sleep on the study schedule. One said, “because there was nothing else . . . so after a while . . . dozed off.” Some reported feeling better rested, having more energy, and reported “a positive effect.” One participant mentioned, “at work, I think I did better actually, as far as feeling more awake.”

## Discussion

Shifting the sleep episode between night shifts to the afternoon evening was feasible according to the majority of participants in both the screening survey and the field trial. Spending 8 hours TIB, either in the afternoon-evening or at a self-selected time, effectively increased sleep duration as compared to the control condition. However, when participants were queried in focus groups about the acceptability of the 8-hour afternoon-evening schedule, despite reporting that they indeed slept longer on the schedule, most reported that family responsibilities and other activities of daily living would make it unsustainable.

The feasibility of an 8-hour afternoon-evening sleep episode was previously demonstrated in a simulated shift work study [11]. This study corroborated and extended those results. Although nearly three-quarters of the participants in the screening survey indicated that they could arrange their schedule to spend 8 hours in bed during the afternoon-evening, the ability to do so depended on demographic and lifestyle factors. Approximately 60% of women, non-Hispanics, and those with primary care of children reported the ability to arrange their schedule to accommodate an 8-hour afternoon-evening sleep episode, as compared to almost 90% of males and Hispanics. In a study of railroad workers (98% male), diary and actigraphy data showed two-thirds of those who slept in one episode following night shifts delayed their sleep to the afternoon-evening [24]. Gender, domestic obligations, and culture have been reported to be related to shiftwork tolerance [25]. Some studies have shown that female shift workers have more difficulty falling asleep and shorter sleep durations, and shift-working women with children have a higher risk of work injury [26, 27]. Perhaps the limited flexibility to allocate 8 hours of sleep in the afternoon-evening is due to the fact that they have primary responsibility for childcare. These responsibilities may contribute to women and those with primary childcare responsibilities exhibiting less tolerance for night work.

Sleep deficiency is highly prevalent in our society, with one-third of US adults reporting that they sleep less than 7 hours per night [28]. In order for adults to obtain the recommended 7–9 hours of sleep each night [29], it is reasonably necessary to spend at least 8 hours in bed to account for sleep latency and nighttime awakenings The healthcare industry has a high prevalence of short sleep duration compared with other industries [30]. Intensive care workers in a recent study averaged less than 6 hours of sleep between nightshifts [31], similar to the duration in the baseline segment and the control group in the intervention segment of this study. Our results demonstrate that spending 8 hours in bed increased sleep duration by about an hour. Results from this study as well as our previous laboratory study [11] suggest that worker education programs that stress the importance of sleep duration for health, combined with instructions to return to bed after awakening and attempt to go back to sleep, are likely to increase sleep duration in older shift workers.

We also investigated the timing of the sleep episodes between night shifts. Sleep propensity is dependent on the interaction of a homeostatic sleep drive and the circadian timing system such that the time of day with the greatest likelihood of obtaining 8 hours of consolidated sleep occurs when bedtime is in the late evening [32]. Despite the reduced propensity for consolidated sleep in the morning hours, most participants randomized to the self-selected 8-hour TIB condition chose to go to bed in the morning following their night shift and increased their sleep duration to an average of 7.1 hours. Although there was an increase in sleep propensity in the early afternoon when the afternoon-evening group was instructed to go to bed, participants in this group averaged only 6.4 hours of sleep, despite spending 8 hours in bed. Although it is important to consider that the propensity for sleep varies with circadian phase, it is often difficult to pinpoint the circadian phase of night shift workers, who have highly variable schedules from day to day and who typically invert their schedules on free days [12]. Moreover, our current study showed that spending 8 hours in bed increased sleep duration regardless of the time of day that sleep was initiated, as we found during our previous simulated shift work study [11].

One of the sleep strategies many night shift workers use is to sleep in two episodes between night shifts [24, 33]. Although the afternoon-evening sleep countermeasure has the advantage of limiting wakefulness prior to the next night shift, a two-sleep episode strategy allows recovery immediately after the shift, and the follow-on nap limits the duration of wakefulness prior to the next shift. Our results indicate the importance of spending 8 hours in bed to obtain adequate sleep. Further studies should explore whether breaking the 8 hours into two blocks achieves the same increases in sleep duration, and whether reducing the duration of wakefulness before starting the night shift improves performance at work.

Despite the effectiveness of the 8-hour TIB countermeasure to increase sleep duration, it was not found to be acceptable to most of the participants. In the study, participants expressed frustration with having to stay in bed as the protocol demanded when they could not sleep. During their normal life, they would likely just get out of bed if they could not sleep or if they had other responsibilities to tend to. The factors in the screening survey that were shown to be predictive of the ability to spend 8 hours in bed during the afternoon-evening were corroborated in the focus groups. The participants, who were mostly women, prioritized childcare and family responsibilities, and other activities of daily living over their need for sleep.

This study had a few limitations. Participants might have correctly assumed that endorsement of being able to spend 8 hours in bed during the afternoon-evening was a study prerequisite. Therefore, the feasibility of that countermeasure might be overestimated in the screening survey. Furthermore, because most participants want to be compliant when being compensated for being in a trial, the feasibility of spending 8 hours in bed between night shifts might be overestimated in the field trial. For the most robust analysis, we chose to use all available data, even when participants did not complete diaries and actigraphy on the same days. However, this limited our ability to address the issue of sleep efficiency. Finally, we only studied older shift workers in the healthcare industry which may limit generalizability to all shift workers.

Obtaining adequate sleep is critical for optimal and safe performance at work and while commuting to, and especially from work [34–36]. Given that older workers represent the fastest-growing segment of the US workforce and that older individuals have a reduced ability to sleep during the daytime compared to younger individuals, it is important to develop effective countermeasures that are feasible and acceptable to this age group [37]. Healthy sleep interventions for shift workers need to address the complexities of childcare, familial, and social responsibilities as these often represent barriers to acceptability and feasibility. Future studies should address personalized strategies to prioritize sleep amongst the many other responsibilities and activities of night shift workers, as well as whether the strategies have any impact on night shift work performance.

## Supplementary Material

zpae010_suppl_Supplementary_Tables_S1

## Notes

This paper is part of the Sleep and Circadian Health in the Justice System Collection.
