# Supplementary material for: Feasibility, effectiveness, and acceptability of an afternoon-evening sleep schedule in older nightshift workers
Source: Sleep Adv. 2024 Feb 7;5(1):zpae010. doi: 10.1093/sleepadvances/zpae010 (PMC10894508; doi:10.1093/sleepadvances/zpae010)
Supplement: zpae010_suppl_Supplementary_Tables_S1 [file zpae010_suppl_supplementary_tables_s1.docx]

Feasibility, Effectiveness and Acceptability of an Afternoon-Evening Sleep Schedule

in Older Nightshift Workers

Laura K. Barger, PhD 1,2

Yuan Zhang, PhD, RN 4

Heidi M Lammers-van der Holst, PhD 1,2,3

Davina Snoep, BSc3

Audra S Murphy 1

Brian Desnoyers, MD, MPH, MS 1, 5

Jeanne F. Duffy, MBA, PhD 1,2

1 Division of Sleep and Circadian Disorders, Department of Medicine, Brigham Women's Hospital, Boston, MA

2 Division of Sleep Medicine, Harvard Medical School, Boston, MA, USA

3 Present address: Department of Public Health, Erasmus University Medical Center, Rotterdam, the Netherlands

4 Solomont School of Nursing, Zuckerberg College of Health Sciences, University of Massachusetts Lowell, Lowell, MA 01886, USA

5 Present address: University of Massachusetts T.H. Chan School of Medicine, , 55 Lake Avenue North, Worcester, MA 01655, USA

Corresponding Author:

Laura K. Barger, PhD

Brigham and Women’s Hospital

Division of Sleep and Circadian Disorders

221 Longwood Avenue, BLI 438

Boston, MA 02115

LKBarger@HMS.Harvard.edu

**Supplement Table 1**

Time in Bed (Mean ± SD, minutes)

|  | **Control** | **8-hr Self-Selected Sleep** | **8-hr Afternoon-Evening Sleep** |
| --- | --- | --- | --- |
| Baseline | 401.5 ± 104.7 | 343.3 ± 104.9 | 383.3 ± 73.8 |
| Intervention | 367.8 ± 102.1 | 471.9 ± 36.8 | 475.4 ± 12.8 |

Sleep (Mean ± SD, minutes)

|  | **Control** | **8-hr Self-Selected Sleep** | **8-hr Afternoon-Evening Sleep** |
| --- | --- | --- | --- |
| Baseline | 372.8 ± 69.1 | 356.4 ± 43.6 | 331.9 ± 75.7 |
| Intervention | 338.0 ± 68.7 | 428.0 ± 66.7 | 386.94 ± 53.2 |
